# Infection with a Nonencapsulated *Bacillus anthracis* Strain in Rabbits—The Role of Bacterial Adhesion and the Potential for a Safe Live Attenuated Vaccine

**DOI:** 10.3390/toxins10120506

**Published:** 2018-12-01

**Authors:** Itai Glinert, Shay Weiss, Assa Sittner, Elad Bar-David, Amir Ben-Shmuel, Josef Schlomovitz, David Kobiler, Haim Levy

**Affiliations:** Department of Infectious Diseases, Israel Institute for Biological Research, P.O. Box 19, Ness Ziona 74100, Israel; itaig@iibr.gov.il (I.G.); shayw@iibr.gov.il (S.W.); assas@iibr.gov.il (A.S.); eladb@iibr.gov.il (E.B.-D.); amirb@iibr.gov.il (A.B.-S.); yossis@iibr.gov.il (J.S.); dkobiler@gmail.com (D.K.)

**Keywords:** *Bacillus anthacis*, vaccine strain, BslA, cell adherence, encephalitis, CNS infection

## Abstract

Nonencapsulated (∆pXO2) *Bacillus anthracis* strains are commonly used as vaccines and for anthrax research, mainly in the mouse model. Previously, we demonstrated that the infection of rabbits, intranasally or subcutaneously, with the spores of a fully virulent strain results in the systemic dissemination of the bacteria, meningitis, and death, whereas ∆pXO2 strains are fully attenuated in this animal model. We used the intravenous inoculation of rabbits to study the pathogenicity of the ∆pXO2 strain infection. Bacteremia, brain bacterial burden, and pathology were used as criteria to compare the Vollum∆pXO2 disease to the wild type Vollum infection. To test the role of adhesion in the virulence of Vollum∆pXO2, we deleted the major adhesion protein BslA and tested the virulence and immunogenicity of this mutant. We found that 50% of the rabbits succumb to Vollum∆pXO2 strain following i.v. infection, a death that was accompanied with significant neurological symptoms. Pathology revealed severe brain infection coupled with an atypical massive bacterial growth into the parenchyma. Contrary to the Vollum strain, deletion of the *bslA* gene fully attenuated the ∆pXO2 strain. Though the Vollum∆pXO2 cannot serve as a model for *B. anthracis* pathogenicity in rabbits, deletion of the *bslA* gene prevents central nervous system (CNS) infections, possibly leading to the generation of a safer vaccine.

## 1. Introduction

*Bacillus anthracis* is the etiological cause of anthrax. It is a gram-positive spore-forming bacillus, naturally infecting herbivores in farms and wildlife [1,2]. Historically, humans contract anthrax by contact with diseased animals and consumption of contaminated animal products, usually leading to contact of spores or bacteria with skin lesions or the digestive tract, respectively [3]. Cutaneous infections lead to 20% to 30% mortality rates, while gastrointestinal infections are almost always lethal [4]. The third and rarest route of infection is spore inhalation [3], rarely documented in natural outbreaks of the last six decades [5,6]. Nevertheless, inhalational anthrax was the major cause of death in the last two major events of spore release in the United States [7] and Soviet Union [8], be it intentional or accidental release. *B. anthracis*’ pathogenicity depends on the presence of two major classes of virulence factors, toxins and capsule, encoded by the pXO1 and pXO2 plasmids. pXO1 encodes the tripartite toxin, Lethal Factor (LF), Edema Factor (EF), and the transport protein Protective Antigen (PA). Binding to a receptor on the mammalian cell membrane, PA is processed by a membrane-bound protease (furin) and forms a heptamer. This heptamer binds three LF and/or EF units, usually in a 2:1 ratio favoring LF. The PA–toxin complex undergoes phagocytosis. Subsequent fusion of the phagosome with a lysosome leads to acidification and PA conformational changes, resulting in the injection of the LF and EF units into the cytoplasm. In the cytoplasm LF, a specific metalloprotease inactivates the MAP kinase regulation pathway, while the calmodulin-dependent adenylate cyclase EF increases intracellular cyclic AMP concentration [9,10]. These activities disrupt normal cellular functions and result in various responses, from immune cell inactivation to cell death. The antiphagocytic poly-γ-D-glutamic acid capsule of *B. anthracis* is encoded by pXO2 and serves as physical protection for the bacterium from host immune responses [11,12]. Though production of poly-γ-glutamic acid is common to other nonpathogenic bacilli, *B. anthracis* is the only family member that binds the polymer to the bacterial cell wall via the activity of the unique gamma glutamyl transferase (ggt)—CapD. CapD activity consists of the general polymer hydrolysis ability that is common to other bacilli ggts, in addition to the sortase activity that catalyzes the binding of the polymer to the cell wall [13]. The CapD is also involved in removal of the capsule polymer from the cell, a process that was suggested to have an effect on the host immune response and as such could be involved in pathogenicity [14]. Since in most cases the infectious form of *B. anthracis* is the spore, the concerted activity of both capsule and toxins are critical for successfully establishing an infection. Endocytosis by phagocytes is the first step in spore-based infection, and prompt capsule production is critical for the germinating bacteria’s survival [10,15,16]. At this stage, toxin secretion is intracellular, in contrast to the later systemic phase where the toxins are secreted into the bloodstream, requiring them to penetrate the host cell from the outside. Disabling capsule production renders the bacteria attenuated, allowing the development of ∆pXO2 (cap^−^) strains, like Sterne, as attenuated live vaccines for livestock and (mainly in Russia and China) humans. Starting in the 1960s, this had a major role in dramatically reducing the global extent of the disease [17,18,19]. It was later established that protection was the result of neutralizing αPA antibodies, which is the key ingredient of the protein-based vaccine.

Since wild-type *B. anthracis* is a BSL3 agent, its research is restricted to adequately equipped institutions (https://www.selectagents.gov/ohp-app1.html). Despite the fact that ∆pXO2 strains are fully attenuated in humans and large animals (livestock), these strains remain lethal in small research animals, like mice and Guinea pigs [20,21]. The fact that these strains produce toxins and induce lethal anthraxlike infections in mouse models, along with their categorization as BSL2 agents, make them a popular surrogate system for studying *B. anthracis* pathogenicity [22]. Previously, we studied the pathogenicity of a fully virulent Vollum strain of *B. anthracis* in rabbits and Guinea pigs [23], and demonstrated that, in these two models, meningitis could be detected in animals that succumbed to the infection [24,25,26], as it was previously only reported for humans and nonhuman primates (NHP) [27,28,29]. This meningitis was toxin-independent, but depended on the presence of the pXO1-encoded regulator atxA [24,25]. In addition, we demonstrated in rabbits that, although subcutaneous (s.c.) spore inoculation of ∆pXO2 strain did not cause any significant symptoms, intravenous (i.v.) injection of vegetative bacteria was lethal [25]. Herein, we characterize the disease that is caused by a ∆pXO2 strain in rabbits and the role of the major pXO1-encoded adhesion protein BslA [30] in this process. We also demonstrate the potential of the ∆*bslA* mutant as a safe live attenuated vaccine in rabbits.

## 2. Results

### 2.1. Pathogenicity of Vollum∆pXO2 Strain in Rabbits Following Different Infection Routes

**Figure 1:**
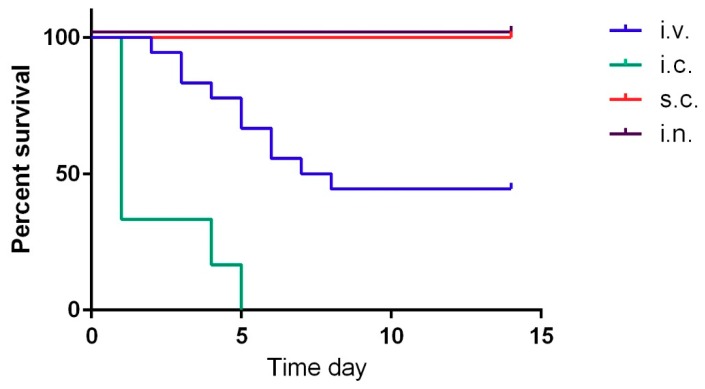
Virulence of the Vollum∆pXO2 by different infection routes. Rabbits were infected with spores subcutaneously (s.c.) (2 × 10^7^ CFU), in red, intranasally (i.n.) (2 × 10^7^ CFU), in black, or vegetative bacteria intravenously (i.v.) (1 × 10^7^ CFU) in blue or intracranially (i.c.) (1 × 10^5^ CFU), in green. Survival of the infected animals was monitored for 14 days. Results represent a single experiment for s.c. and i.n., three independent experiments for the i.v., and two independent experiments of the i.c. inoculation.

**Figure 2:**
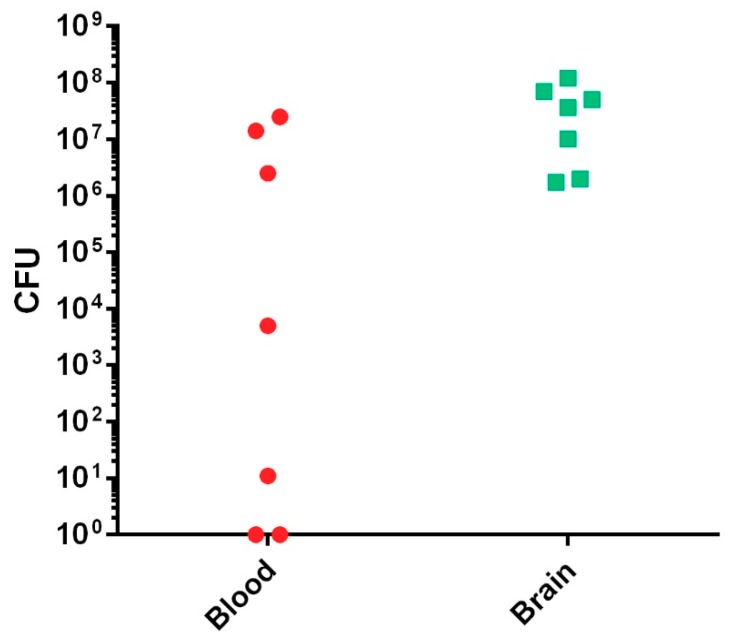
Postmortem bacteremia and brain bacterial burden in rabbits that succumbed to the i.v. Vollum∆pXO2 infection. Blood samples and whole brain tissue were collected postmortem. Bacteremia (CFU/mL) was determined by serial dilution and plate colony counting. Organ homogenization and plate counting determined brain bacterial burden (CFU/organ).

Since the Vollum∆pXO2 is similar to the Sterne vaccine strain, we tested the pathogenicity of this strain by high-dose spore infection. As expected, this strain was completely attenuated when 2 × 10^7^ CFU of spores were administered via intranasal (i.n.) or subcutaneous (s.c.) routes (Table 1, Figure 1). Since this strain is missing the antiphagocytic capsule, a major virulence factor, we tested the virulence of this strain by intravenous (i.v.) injection of vegetative bacteria. As we previously demonstrated, this route of infection bypasses the initial incubation steps that normally include spore germination and migration to a lymph node, delivering the bacteria directly into the blood stream, generating bacteremia [25]. An injection of 10^7^ CFU of Vollum∆pXO2 bacteria resulted in the death of slightly more than 50% of the rabbits, as was determined in three independent experiments with similar outcomes (Table 1, Figure 1). Unlike the death of the rabbits from the wild-type (WT) Vollum strain, Vollum∆pXO2 showed a significantly longer mean time to death (MTTD) (Table 1, Figure 1) while also exhibiting significant neurological symptoms such as tilted head, uncontrolled eye movement, and disorientation. The neurological symptoms were detected mainly in animals that succumbed from day 5 onward. In order to explore the pathogenic mechanism of the Vollum∆pXO2 strain, we determined the bacterial concentration in the blood (bacteremia) and brains of rabbits that succumbed to i.v. infections. While the bacteremia level varied from nondetectable to 10^7^ CFU/mL, brain bacterial load was high in all tested animals, in the range of 10^7^ CFU/organ (Figure 2). To explore the possibility that central nervous system (CNS) infection correlates with death from Vollum∆pXO2, we tested the outcome of intracranial inoculation (i.c.). Injecting the Vollum∆pXO2 strain i.c. resulted in 100% lethality, with an MTTD slightly longer than that of the WT strain, 2.1 days compared to 1 day, as was demonstrated in two independent experiments (Table 1, Figure 1).

### 2.2. Brain Pathology of the Vollum∆pXO2 Infection

**Figure 3:**
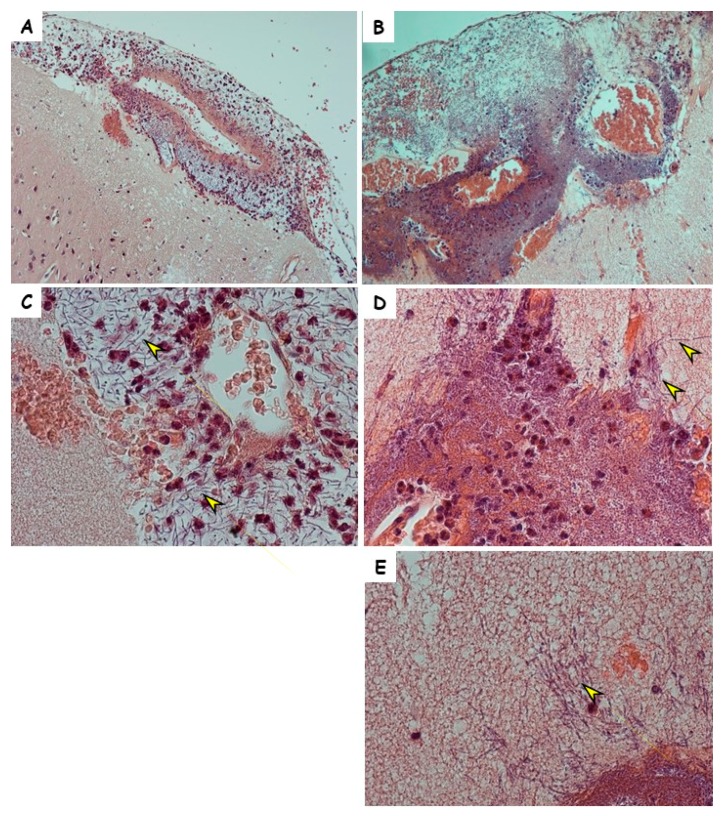
Pathology of representative brains from rabbits that were infected using Vollum or Vollum∆pXO2 strains. Hematoxylin and Eosin staining of brain tissue from rabbits that died following i.n. infection with (**A**,**C**) the wild-type Vollum strain or (**B**,**D**,**E**) i.v. injection of the Vollum∆pXO2 strain. Image magnification ×100 (**A**,**B**) and ×400 (**C**–**E**). Bacilli are marked with arrow heads.

**Figure 4:**
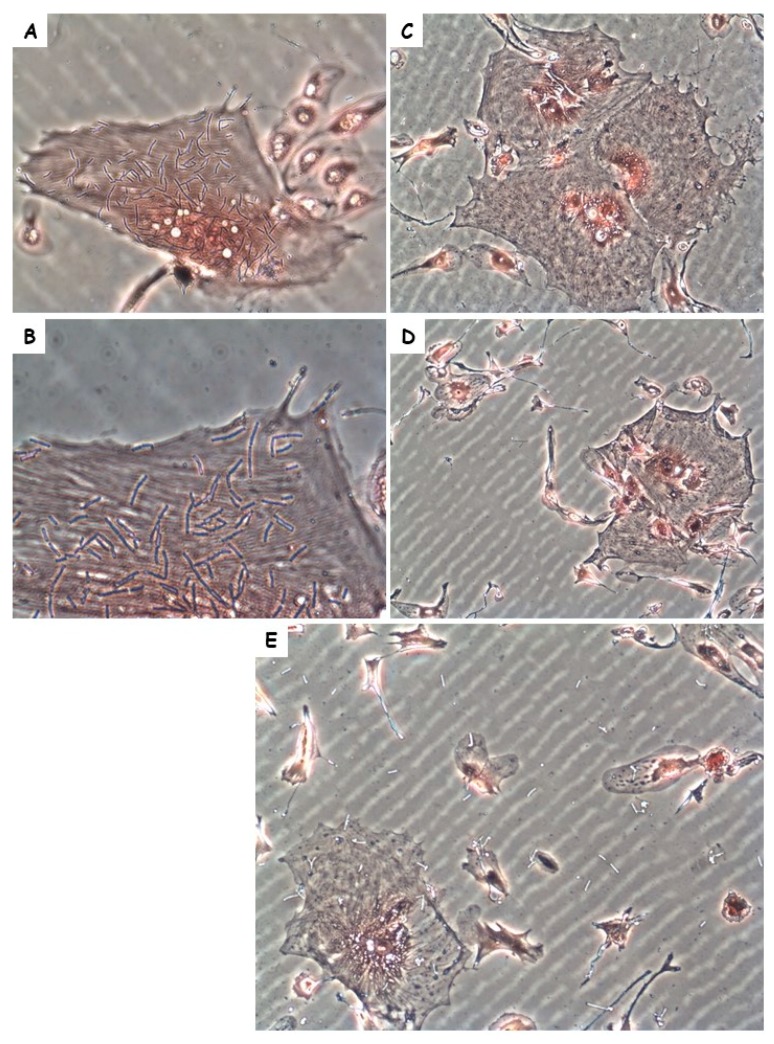
Adhesion of the Vollum or Vollum∆pXO2 strains to brain cell cultures. Adhesion test of (**C**–**E**) encapsulated Vollum or (**A**,**B**) Vollum∆pXO2 bacteria to rabbit brain cell cultures. Image magnification ×200 for (**A**,**C**–**E**) and ×400 for (**B**).

Meningitis is a part of anthrax pathology in rabbits [24,26]. Since the symptoms in the Vollum∆pXO2-inoculated rabbits were indicative of CNS infection, we performed histopathological analysis of the brain sections of three of the 10 rabbits that succumbed to the i.v. inoculation. Anthrax-induced CNS infections are typically meningitis, i.e., bacterial growth in the meninges, particularly in the CSF, without any spread into the brain parenchyma (for example, Figure 3A,C). Intravenous infection with Vollum∆pXO2, on the other hand, results in massive damage to the brain cortex (Figure 3B) with what appears to be massive bacterial growth. Higher-magnification examination (Figure 3D,E) revealed bacterial growth into the parenchyma, i.e., encephalitis, of the nonencapsulated bacteria. One of the major differences between the encapsulated and no-encapsulated strains is in their ability to adhere to cell tissue. To examine if such difference exists in the case of rabbit brain cells, we tested the adherence of capsular Vollum and noncapsular Vollum∆pXO2 to cultured brain cells. The bacterial cells were cultured in DMEM 10% FCS in 10% CO_2_ to induce the production of virulence factors (toxins and capsule) prior to the adherence test. The adherence test clearly demonstrated (Figure 4) that, while the nonencapsulated Vollum∆pXO2 strain efficiently adheres to the cells (Figure 4A,B), the WT-encapsulated strain does not adhere at all (Figure 4C,D). Equal nonspecific adherence of the WT to the surface and the cells was observed in some of the tests (Figure 4E). Since no significant differences were observed in the bacterial adhesion to the plate surface and the cells, we conclude that this nonspecific adherence is probably an artifact and not true cell adhesion.

### 2.3. Role of BslA Adherence Protein in the Pathogenicity of Vollum∆pXO2

BslA is a pXO1-encoded S-layer protein that is regulated by AtxA, a major virulence regulation factor [30]. The major role played by BslA in adherence to the Sterne strain (∆pXO2) to a variety of cell lines was previously demonstrated [31,32]. Deleting this protein lead to the loss of the bacteria’s adherence capabilities. Since CNS infection requires adherence of the bacteria to the endothelial cells prior to crossing the blood–brain barrier (BBB), we used genetic tools to test the possible role of the BslA protein in Vollum∆pXO2 virulence in rabbits. The effect of complete deletion of the *bslA* (formally known as pXO1-90) gene on the virulence of the Vollum∆pXO2 strain was tested by i.v. injection of 2 × 10^7^ CFU of vegetative bacteria. All the rabbits survived the infection without any detectable clinical signs (Table 2), indicating that adherence via BslA is crucial for successful infection by Vollum∆pXO2 strain.

### 2.4. Vaccine Potential of the Vollum∆pXO2∆bslA Strain

**Figure 5:**
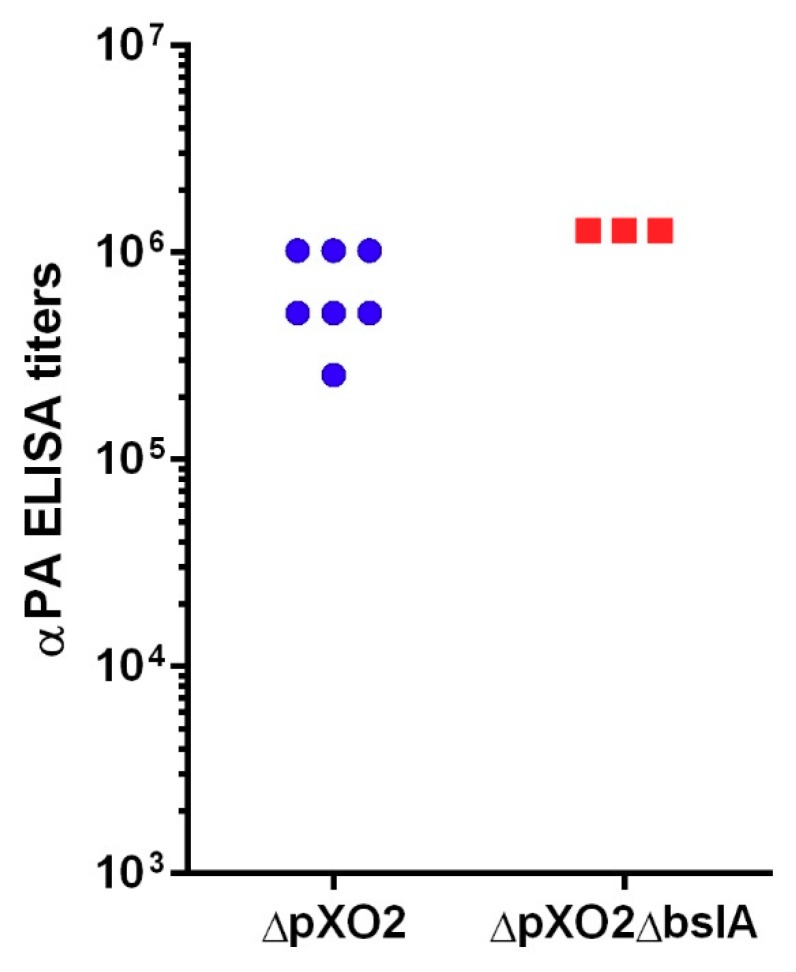
Total αPA ELISA titers in rabbits that were infected i.v. with Vollum∆pXO2 or Vollum∆pXO2∆bslA. Total αPA-specific antibodies were determined in the serum of rabbits infected i.v. with Vollum∆pXO2 (blue circles) or Vollum∆pXO2∆bslA (red rectangles). Units are E-1 of the highest serial dilution that presented a signal-to-noise ratio of at least 2.

The Vollum∆pXO2 strain is genetically similar to the Sterne vaccine strain used in the vaccination of livestock. It is also similar to the Sterne-like human vaccines used in Russia and China. By injecting vegetative bacteria directly into the bloodstream, we induced an artificial disease that could progress into two distinct scenarios: either the bacteria succeed in infecting the CNS early on, leading to a fatal disease, or the progression is slower, instead leading to rapid clearance. We evaluated the possibility that the i.v. injection of the Vollum∆pXO2, when the animals survive the infection leads to effective subsequent vaccination. We tested the specific total αPA antibodies titers in sera from the surviving animals. The results in Figure 5 demonstrate that all the rabbits that survived the i.v. infection developed high αPA antibodies titers of 2.6 × 10^5^ to 1.2 × 10^6^. Deleting *bslA* in the Vollum∆pXO2 strain resulted in full attenuation of this strain by the i.v. infection route. Therefore, we tested specific αPA antibodies sera titers in animals that received i.v. injections with the Vollum∆pXO2∆*bslA* mutant in order to check if the additional deletion affected the strain’s immunogenicity. The results in Figure 5 clearly demonstrate that, while the *bslA* mutation completely attenuated the ∆pXO2 Vollum strain, it did not have any negative effect on the immunogenicity of the mutant, with rabbits presenting αPA titers of 1.2 × 10^6^ that were similar to the original Vollum∆pXO2 strain. To test the ability of this “vaccination” to confer protective immunity, two weeks after i.v. inoculation with the mutant strain the rabbits were challenged s.c. with 10^3^ CFU spores of the fully virulent Vollum strain (50 LD_50_). All the animals survived this challenge without any observable signs of illness (Table 2). Since the Sterne vaccine is administered as a s.c. injection of spores, we tested the ability of the Vollum∆pXO2∆*bslA* to generate protective immunity by a single s.c. spore injection. Rabbits were immunized by s.c. injection of 2 × 10^7^ CFU of spores, and 14 days afterwards challenged with 10^3^ s.c. injection of Vollum spores. All the animals survived (Table 2) the 50 LD_50_ challenge, indicating that the ∆*bslA* mutant conferred full protection in rabbits following a single dose of 2 × 10^7^ CFU of spores administrated subcutaneously.

## 3. Discussion

Sterne-like strains have a major role in anthrax prevention in farm animals and humans, as well as in *B. anthracis* research. Strains lacking pXO2 are attenuated, partly in the mouse and Guinea pig models, and completely in larger animals such as rabbits, NHP, and cattle [20]. We took advantage of our systemic infection model to examine the pathogenicity of the Vollum∆pXO2 strain in the rabbit model, which is one of the two recommended animal models for *B. anthracis* research. We demonstrated that i.v. injection of 2 × 10^7^ of vegetative bacterial cells resulted in the death of more than 50% of the infected rabbits (Figure 1, Table 1). We also observed significant symptoms of brain damage, indicating CNS infection. This CNS infection was more severe compared to the wild-type Vollum strain, probably due to the longer course of disease progression till death.

To explore the possibility that death from the ∆pXO2 was correlated with CNS infection, we demonstrated that injecting bacteria i.c. resulted in the death of all the infected animals. In this type of infection, the time to death was slightly longer compared to the wild-type strain (2.1 versus 1) in this type of infection (Figure 1, Table 1). CNS infection as part of anthrax was previously documented in humans, NHP, rabbits, and Guinea pigs following systemic disease resulting from infections with fully virulent anthrax strains. In humans and NHP, the common finding was extensive cerebral hemorrhages while in the Guinea pig and rabbit models, the hemorrhage was less severe. However, meningitis could be detected histologically in all models. Histopathology of brains from rabbits that died following the Vollum∆pXO2 infection exhibited a distinct type of infection (Figure 3). In this case, on top of the bacterial growth in the meninges (meningitis), a significant growth into the brain parenchyma could be detected (encephalitis). This type of growth is not typical to WT *B. anthracis* infections [26,29] and could be due to the absence of the typical poly γ-d-glutamic acid capsule in this strain (∆pXO2). In this strain, the surface of the bacterial cell is exposed, enabling direct noninterrupted interactions of the S-layer proteins with the host tissues [32].

To explore the possibility of differences between the encapsulated and nonencapsulated strains, we tested the ability of these strains to adhere to host cells. Unlike the encapsulated wild-type bacteria, the ∆pXO2 strain adhered to cells in culture. This adherence was differential and it appeared that the bacteria adhered more efficiently to some types of cells compared to others (Figure 4). A crucial property for CNS invasion is adherence capability, in this case mediated by BslA, a protein that under pathogenicity-inducing conditions consists about 30% of the S-layer proteins [32]. The effect of *bslA* deletion on adhesion of Sterne strain to endothelial cell was previously reported [31,32]. Deletion of *bslA* in the Vollum∆pXO2 strain resulted in full attenuation (Table 2). We previously demonstrated that deletion of *bslA* in wild-type Vollum strain had little effect on virulence in the rabbit model, if any [25]. This could be the result of protein-masking by the bacterial capsule. We therefore proposed that wild-type bacteria utilize host responses, for example, coagulation [26], for adhesion. The fact that in the absence of BslA the ∆pXO2 is fully attenuated and the assumption that death is a result in CNS infection emphasizes the importance of adhesins in the ability of this strain to cross the blood–brain barrier.

∆pXO2 strains are used in the Western world as live attenuated vaccines for farm animals. These vaccines are based on i.m. injection of spores with protection correlated mainly to immune reactions against the protective antigen, i.e., αPA-neutralizing antibodies. Since PA is made and secreted by vegetative bacterial cells during infection, the fact that the host develops a protective immune response indicates that spores germinate and invade the bloodstream, however transiently. Generating bacteremia using the ∆pXO2 strain by i.v. injection results in death or immunity, probably depending on host immune responses. The fact that, in the absence of BslA, this artificial bacteremia generates only protective immune responses (Figure 5, Table 2) indicates that the mutant manages to survive for a sufficient period of time in the bloodstream to induce protective immunity. The ability of the mutant to confer immunity in response to a single s.c. spore injection (Table 2) suggests that the main effect of the *bslA* mutation is mainly on CNS infection rather than the first steps of host invasion. These findings make this mutant a perfect candidate for a safer agricultural live attenuated vaccine.

As a lethal and rare disease, anthrax research depends on epidemiological studies and experiments performed in animal models [33]. Since *B. anthracis* is a Tier 1 agent and restricted to BSL3 facilities (https://www.selectagents.gov/ohp-app1.html), there is a limited number of laboratories that can use the fully virulent strain in their research. Most of these laboratories are affiliated with governments. Sterne-like strains are exempted from the Tier 1 restrictions (https://www.selectagents.gov/exclusions-overlap.html#bacillus) and are classified as BSL2, enabling anthrax-related research in most academic institutes. Since Sterne-like strains are attenuated in most relevant animal models (i.e., rabbit and NHP) most Sterne studies are done in mice [20]. Animal models, by definition, serve as a model for human diseases, and each animal model has its pros and cons [22]. In rabbits, comparing the disease caused by the wild-type and attenuated strains, we found similarities between Vollum and Vollum∆pXO2 in the development of a CNS infection. However, pathology is significantly different, with meningitis in the Vollum strain infection [24] complemented with encephalitis in the Vollum∆pXO2 case (Figure 3). The importance of bacterial adhesion to *B. anthracis* pathogenicity is similar for the Vollum [26] and Vollum∆pXO2 strains. However, while the deletion of the *bslA* gene completely attenuates Vollum∆pXO2 (Table 2), it has a minor to no effect on the pathogenicity of the Vollum strain, as it uses an alternative adherence mechanism [25]. Therefore, though we cannot learn from the Vollum∆pXO2 studies on *B. anthracis* pathogenicity, we can conclude that, in rabbits, the virulence of the Sterne-like vaccine depends on their being able to cross the blood–brain barrier. Hence, a way to prevent this CNS infection of the ∆pXO2 strain and generate a safer vaccine would be to delete the *bslA* gene.

## 4. Materials and Methods

### 4.1. Bacteria Strains, Media, and Growth Conditions

In this study, we used the following *B. anthracis* strains: Vollum (ATCC 15578) from the IIBR collection, and Vollum∆pXO2 [23,24]. Sporulation was carried out as previously described, using a G broth [34]. The disruption of the *bslA* gene was performed by homologous recombination, as previously described [35]. In general, gene deletion was accomplished by a markerless allelic exchange, replacing most of gene-coding region with the SpeI restriction site. The resulting mutant did not code for any foreign sequences. The primers that were used for the deletion: 2: gacgcgcggccgcaggatatgcccacg and 3c: ggactagtgcgttttctctgtgtgc (54 nucletids upstream of the first AUG) and 4: ggactagtgtaaccctaaacc and 5c: ttggcgcgcccatatataatagtacctcc (178 nucleotides upstream of the termination codon). The mutation was verified by PCR.

### 4.2. Rabbit Infections

Female New Zealand white rabbits (Charles River Laboratories, 2.2–2.5 kg) were infected with wild-type and mutant Vollum strains. Germination of Vollum∆pXO2 and Vollum∆pXO2∆*bslA* spores was done by incubation in Terrific broth for 30 min, followed by 2 h incubation in DMEM-10% FBS in order to induce toxin secretion in a CO_2_ incubator (10% CO_2_). Vegetative bacteria were injected i.v. to rabbits. Rabbits were infected with Vollum or mutant Vollum∆pXO2∆*bslA* spores. For spore infections, spore preparations were heat-shocked (70 °C, 20 min) prior to infecting the animals. Serial dilutions in saline were then performed to achieve spore suspensions of 10^3^ CFU/mL. A dose of 1 mL was administered subcutaneously (s.c.) to each animal. Animals were followed for 14 days (daily inspections) or for as long as otherwise mentioned. Blood samples from deceased animals were plated. Grown bacteria was subjected to DNA extraction and PCR analysis to determine the strain responsible for the animals’ death.

This study was carried out in strict accordance with the recommendations of the Guide for the Care and Use of Laboratory Animals of the National Research Council. All protocols were approved by the Committee on the Ethics of Animal Experiments at the IIBR, permit numbers RB-20-14 (23 October 2014), RB-01-16 (14 January 2016), and RB-04-16 (28 January 2016). The following conditions were defined as endpoints, with animals euthanized upon displaying one of them: severe respiratory distress or the loss of the righting reflex. Euthanasia was performed using sodium pentobarbital injections. Differences between MTTD were determined by using Mann–Whitney and t-tests in GraphPad Prism version 5.00 (GraphPad Software, San Diego, CA, USA, www.graphpad.com).

### 4.3. Determination of Tissue Bacterial Burdens

Infected rabbits were euthanized 5 or 24 h postinfection with sodium pentobarbital, followed by organ harvest. The brain, was removed, promptly homogenized and serially diluted and plated on solid plates to enumerate bacterial levels.

### 4.4. Isolation of Rabbit Primary Brain Endothelial Cells (RB-PBEC)

The brain from a freshly sacrificed rabbit was removed and placed in a cold solution of 70% ethanol. The meninges were manually and aseptically removed. The brain (~10 mL in volume) was then coarsely homogenized with 5 mL of M-199 medium (Biological Industries, Beit-Haemek, Israel). The lysate was then supplemented with 250 mg of Dispase-II powder (Sigma, Tel Aviv-Yafo, Israel), followed by 2 h incubation at 37 °C with gentle agitation on a rotary shaker. After the incubation, 25 mL of 10% dextran solution in M-199 medium (500,000 molecular weight, Sigma, Tel Aviv-Yafo, Israel) were mixed into the lysate and the mixture was centrifuged (7000× *g*, 4 °C, 20 min). The pellet was collected and suspended in 10 mL of 0.16% Dispades-II solution in M-199 medium, then incubated for 1 h at 37 °C with gentle agitation. Following this incubation, the lysate was gently loaded onto a 2-phase ficoll gradient (15 mL upper phase at a density of 1.03, 10 mL lower phase at a density of 1.07), and centrifuged at room temperature, 1250× *g*. The turbid midphase containing the cells was aspirated, washed in complete M-199 growth medium, and plated in a T-75 flask coated with type-I rat tail collagen (Promocell, Sigma, Tel Aviv-Yafo, Israel). The cells were cultured up to 5 passages, and then discarded.

### 4.5. In Vitro Bacterial Adhesion Assay Using RB-PBEC

The cells isolated and cultured as described above were plated on 6-well plates coated with type-I rat tail collagen (Promocell, Sigma, Tel Aviv-Yafo, Israel) and grown for 48–72 h to confluence. To each well, ~10^6^ vegetative bacteria were added and incubated for 3 h. The plates were gently aspirated and washed 6 times with warm PBS, followed by fixation with 10% formaldehyde. The fixed cells were stained using a gram-stain kit (Sigma, Tel Aviv-Yafo, Israel), dried, and photographed under a microscope.

### 4.6. Histopathological Tissue Processing

Immediately following postmortem harvest of selected brains, they were fixed in 50 mL tubes containing ~30 mL of 3.7% formaldehyde (in PBS). After fixation, 4–5 mm thick coronal brain sections were placed in separate histological cassettes. The cassettes were then paraffinized overnight using a Leica APS200 system (Leica Biosystems, Wetzler, Germany). The paraffinized tissue slices were embedded in paraffin blocks, and slides were prepared by mounting 5 μm thick sections cut on a rotary microtome (Leica Biosystems, Wetzler, Germany).

### 4.7. Histopathological Staining

Slides prepared as described above were stained using an optimized Hematoxylin and Eosin staining protocol. Briefly, slides were deparaffinized, then rehydrated in a gradient of ethanol (100%–70%) and finally in water, then stained for 5 min with hematoxylin and 15 min with eosin.

### 4.8. Image Acquisition

Images were taken using a Zeiss Axioskop microscope (Zeiss, Oberkochen, Germany) equipped with a Nikon DS-Ri1 camera controlled by a DS-U3 Digital Sight and the Nis-Elements-Br software suite (Nikon, Tokyo, Japan).

## Figures and Tables

**Table 1:** Virulence of the Vollum∆pXO2 strain in rabbits.

Infection Route	Dose * (WT LD50)	Dead/Infected	Mean Time to Death (MTTD) (Days)
s.c.	2 × 10^7^ (10^6^)	0/4	/
i.n.	2 × 10^7^ (10^3^)	0/4	/
i.v.	1 × 10^7^ (10^6^)	10/18 **	4.9
i.c.	1 × 10^5^ (10^4^)	6/6 ***	2.1

* CFU, in parentheses, LD50 of the Vollum strain; ** the sum of three independent experiments; *** the sum of two independent experiments.

**Table 2:** Virulence and vaccination potential of the Vollum∆pXO2∆bslA mutant in rabbits.

Infection Route	Dose (CFU)	Dead/Infected	Challenge Route	Strain	Dose (CFU)	Dead/Challenged
i.v.	2 × 10^7^	0/4	s.c.	Vollum	10^3^	0/4
s.c.	2 × 10^7^	0/4	s.c.	Vollum	10^3^	0/4
